# Remembering Sets: Capacity Limit and Time Limit of Ensemble Representations in Working Memory

**DOI:** 10.3390/bs13100856

**Published:** 2023-10-19

**Authors:** Chaoer Xu, Yingzhu Qian, Hui Chen, Mowei Shen, Jifan Zhou

**Affiliations:** Department of Psychology and Behavioral Sciences, Zhejiang University, Hangzhou 310058, China

**Keywords:** ensemble representation, working memory, capacity limit, time limit, change detection

## Abstract

In a constantly changing visual environment, the ability to extract and store ensemble representations plays a crucial role in efficiently processing and remembering complex visual information. However, how working memory maintains these ensemble representations remains unclear. Therefore, the present study aimed to investigate the limits and characteristics of ensemble representations in working memory using a change detection paradigm. Participants were presented with multiple sets of circles grouped by spatial proximity and were asked to memorize the mean diameter of the circles in each set. Results showed that working memory could stably maintain mean sizes of approximately two sets for at least four seconds. Moreover, the memory performance of ensembles was not affected by the number of circles within a set, suggesting that individual details were not stored in working memory. These results suggest that the visual system can effectively store ensembles in working memory without preserving detailed individual information.

## 1. Introduction

In our daily life, we rarely encounter objects in isolation. Rather, they tend to appear in groups of the same category, such as a group of people in a park or a row of books on a shelf. Recent studies have shown that the visual system can represent such sets of similar objects by extracting their statistical properties, such as mean and variance [1]. This process is termed ensemble representation, and it has been observed in both low-level features such as size, orientation, and color [2,3,4] and high-level features like facial expression, identity, and group behavior [5,6,7]. Importantly, ensemble summaries are extracted rapidly and efficiently, thereby reducing the burden on the visual system by summarizing complex visual information [8,9].

Several studies have suggested that ensemble encoding is robust to changes in the number of individuals within the set. For example, Ariely [3] found that participants could accurately report the mean diameter of a set of circles, and their performance was unaffected by the number of circles. Similarly, Attarha and his colleagues [10] adopted the simultaneous–sequential method to test the processing capacity for summary statistics of a single ensemble. Four clusters of stimuli, each composed of circles with varying sizes, were presented around fixation, and observers had to identify the cluster with the largest or smallest mean size. In the simultaneous condition, all four clusters were presented concurrently, while in the sequential condition, they appeared sequentially with two clusters at a time. The results showed that the processing capacity was not limited by the number of items in the set. Ensemble perception can also be extracted even if the individuals composing the ensemble cannot be reliably recognized. For example, participants were able to accurately judge the color variance of letters that were not attended to [11]. A study on sets of faces also showed that participants could detect a change in the average expression between two sets of faces, even when failing to localize which specific faces caused the change [12].

However, to date, most previous studies have focused on the perceptual processing of ensemble representation [1,13]; few studies have addressed the processing of ensemble representation in working memory, which is important for the visual system to preserve visual stability. To form a coherent visual perception in dynamic and complex visual scenes, we need to simultaneously retain multiple ensembles in working memory. For instance, imagine walking along a bustling city street. You have to pay attention to and remember multiple groups of stimuli around you, such as people walking and cars driving by. The ability to extract and retain information about these groups is crucial for ensuring your safety and unhindered passage through the bustling street. Therefore, investigating how working memory maintains multiple ensemble representations is critical for understanding the mechanisms underlying our ability to process and navigate complex visual environments.

To this end, the current study examined both the capacity limit and the time limit of ensemble representations in working memory. Previous studies have primarily focused on investigating the capacity limit of spatially intermixed sets, which are collections of items defined by different colors [14,15,16,17]. In the study conducted by Halberda, Sires, and Feigenson [14], participants were presented with arrays of dots of different colors and were asked to enumerate multiple color-defined dot groups. The researchers compared the participants’ performances in the pre-cue condition, where they were informed in advance which subset to enumerate, and the post-cue condition, where they were not told until the array had disappeared. They found that participants were able to enumerate approximately two subsets, along with the superset of all dots. Since participants did not know which subset to enumerate until 500 ms after the array had disappeared on post-cue trials, enumerations needed to be held in working memory during this 500 ms, which indicates that working memory can maintain three enumerations. Similarly, Luo and Zhao [17] employed the pre-cue and post-cue paradigm to investigate the capacity limit of multiple color-defined circle groups. In their study, participants were asked to estimate the mean size of each set and the results showed that participants could accurately estimate mean sizes of up to two sets of circles. These findings suggest that the capacity of ensemble representation in working memory is limited. However, working memory has been found to have not only a capacity limit (i.e., the number of items that can be held) but also a time limit (i.e., how long items can be held before they are forgotten or displaced) [18,19,20,21]. In order to better understand the dynamics of information stored in working memory [22], both the capacity and temporal decay of ensemble representations were measured in this study.

Here, we adopted the change detection paradigm [23] to explore the limits and characteristics of ensemble representations in working memory. This task is commonly used in measuring working memory capacity for visual objects [24], allowing for a comparison of results between ensembles and individual objects. Participants were presented with several sets of circles grouped by spatial proximity and were asked to memorize the mean diameter of each set. We manipulated the number of sets (Experiment 1) and retention interval (Experiment 2) to examine the capacity and time limits of storing ensembles in working memory. To estimate the capacity limit of ensemble representations, this study grouped objects based on spatial proximity instead of color, unlike previous research that measured the capacity limit [14,15,16,17]. This approach allowed us to distinguish the capacity for the set from the capacity for the set’s defining features [25]. Additionally, the use of non-interspersing groups eliminates location-based interference [26], which could have been a potential confounding factor in previous studies that used color as the grouping method. Although some previous studies found that individual information does not affect perceptual ensemble representation, it may be different in working memory. Since some individuals may be sampled and stored in working memory to provide extra information to enrich and correct the representation of ensemble, in this case, individual information may affect the memorized ensembles. Thus, it is necessary to run a working memory experiment to directly test whether the individual information affects memorized ensembles. In Experiment 3, we varied the number of circles in a set to explore whether it influenced the performance of memorizing ensembles. As mentioned above, perceptual studies have shown that the number of elements has little or no effect on ensemble encoding [3,10,27]. To extend previous findings derived from perceptual paradigms, this experiment examined the effect of the number of elements on the memory of ensemble representations in working memory.

## 2. Experiment 1

Experiment 1 first examined working memory capacity for ensemble representation by manipulating the memory load of the set in a change-detection task.

### 2.1. Method

#### 2.1.1. Participants

A priori power analysis was conducted with the program G*Power 3.1 [28]. Given an estimated effect size of Cohen’s *f* = 0.50 (based on the pilot experiment of 10 participants with the same experimental design as Experiment 1) and α = 0.05, a sample size of 16 participants was sufficient to detect an effect with a power of 1 − β = 0.99. Twenty-four Zhejiang University undergraduates (15 females) participated in this experiment. Two participants were removed from further analysis because of overall chance level performance. All participants (13 females, age range of 18 to 24 years) had normal or corrected-to-normal vision, signed informed consent, and received course credit or monetary compensation. The study was approved by the Institutional Review Board at the Department of Psychology and Behavioral Sciences, Zhejiang University.

#### 2.1.2. Apparatus and Stimuli

The stimuli were created using MATLAB and Psychophysics Toolbox software [29] and presented on a 17-inch CRT monitor with a spatial resolution of 1024 × 768 pixels at a 100 Hz refresh rate. Participants maintained a distance of approximately 60 cm from the center of the monitors, and the experiments were conducted in dark rooms.

**Figure 1:**
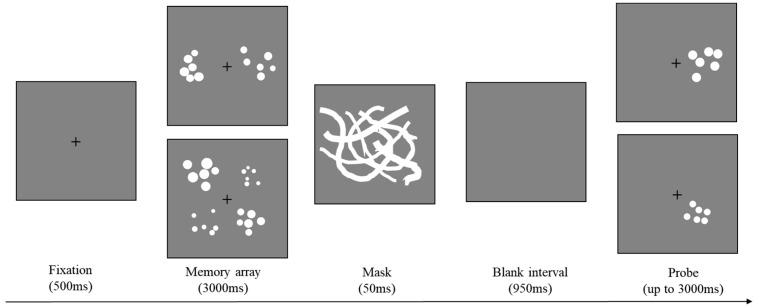
A schematic illustration of a single trial in Experiment 1. Here, a trial with two or four sets of circles is shown. Participants were instructed to memorize the mean diameter of each set.

The memory array consisted of multiple sets of circles (2, 4, or 6), with six circles of different sizes in each set (see Figure 1 for details). To determine the diameters of the circles within each set, we initially established the mean diameter for each set. This mean diameter was randomly selected from eight values, which were equally spaced on a logarithmic scale separated by a factor of 1.28, ranging from 0.43° to 2.41°. After determining the mean diameter, the diameters of individual circles were randomly sampled from a normal distribution in which the mean was the given set mean and the standard deviation was one-fourteenth of its mean. The spatial locations of the sets were evenly distributed on an invisible circle with a radius of 7.14° from the center of the screen.

The mask was a pattern formed by overlaying multiple curves with different widths. Eight values calculated above were chosen as the widths for these curves. Masking was used to prevent interference from the visual afterimage and to avoid iconic memory.

#### 2.1.3. Procedures and Design

Each trial began with a central fixation (see Figure 1) presented for 500 ms to inform the participants of the upcoming memory task. Then the memory array with 2, 4, or 6 sets of circles appeared and lasted for 3000 ms, immediately followed by a mask presented for 50 ms. Participants were instructed to memorize the mean diameter of each set. After a 950 ms blank interval, the participants were presented with a single probe set containing six circles of the same size. The participants had to judge whether the mean diameter of the probe set was the same as the mean diameter of the memory set at that location by pressing a button on the keyboard within 3000 ms. The participants were asked to press “F” if the mean diameter changed and “J” if the mean diameter stayed the same. In 50% of the trials, they had the same mean diameter; in 25% of the trials, the mean diameter of the probe set was 1.28 times bigger than that of the memory set; in the remaining 25% of the trials, the mean diameter of the memory set was 1.28 times bigger than that of the probe set. Response accuracy was emphasized and analyzed. There was a 1500 to 2000 ms blank interval between trials.

A one-factor (the number of sets: 2, 4, and 6 sets of circles) within-subjects design was adopted. The different conditions were tested in a mixed design. Each condition consisted of 60 trials, resulting in a total of 180 trials. The experiment lasted about 40 min, divided into four sessions with 3-min breaks between them. Participants completed a minimum of 16 practice trials before the main experiment to ensure they understood the instructions.

#### 2.1.4. Data Analysis

We report the results below using two main measures: memory capacity estimates and the accuracy of the change detection task.

To estimate the working memory capacity of ensemble representations, we employed Cowan’s formula [23], *K = S × (H − FA)*, where *K* is working memory capacity, *S* is the number of sets, *H* is the hit rate, and *FA* is the false alarm rate. We calculated *K* for each condition of each participant. To have a more accurate estimate, we took the maximum *K (K*-max) of all the conditions as one’s working memory capacity and then used the average *K*-max of all participants as the total working memory capacity [30].

We used *t*-tests (two tailed) and ANOVAs for all statistical analyses. In all ANOVAs reported in this article, we reported the Greenhouse–Geisser-corrected *p*-value if sphericity was violated. Additionally, the Bayes factor was computed using JASP with default priors [31]. We reported the Bayes factor for the alternative hypothesis relative to the null hypothesis, namely BF_10_. A BF_10_ greater than 3 implies moderate support for the alternative hypothesis, while values between 0.33 and 3 suggest weak or anecdotal evidence, and values below 0.33 provide moderate support for the null hypothesis [32,33].

### 2.2. Results

**Figure 2:**
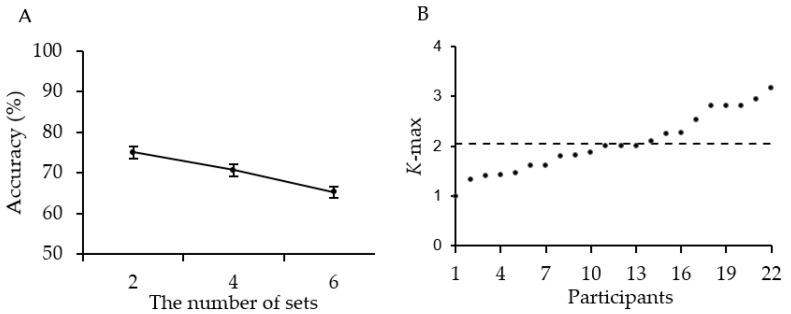
Results of Experiment 1. (**A**) The accuracy of each condition. Error bars represent SEM. (**B**) Each point represents the *K*-max for an individual participant (in increasing order), while the dashed line indicates the average *K*-max across participants, which is 2.04.

The accuracy of the change-detection task is shown in Figure 2A. A one-way repeated-measures ANOVA yielded a significant main effect of the number of sets (*F*(2, 42) = 18.184, *p* < 0.001, $\eta_{p}^{2}$ = 0.464, *BF*_10_ = 17,991.920). Post hoc contrasts revealed that the accuracy was significantly higher at two sets than at both four sets (75% vs. 70%, *t*(21) = 2.673, *p* = 0.014, Cohen’s *d* = 0.570, *BF*_10_ = 3.707) and six sets (75% vs. 65%, *t*(21) = 5.982, *p* < 0.001, Cohen’s *d* = 1.275, *BF*_10_ = 3399.705). The accuracy was also significantly higher at four sets than at six sets (70% vs. 65%, *t*(21) = 3.372, *p* = 0.003, Cohen’s *d* = 0.719, *BF*_10_ = 14.310). This result indicated that the memory accuracy decreased when the number of sets in the memory display increased.

Figure 2B shows participants’ *K*-max and their average, with *K*-max ranging from 1 to 3.17 (*M* = 2.04, *SD* = 0.59, 95% CI = [1.78, 2.3]). The average *K*-max suggested that participants could extract and hold approximately 2.04 mean sizes in working memory. This was consistent with the two-set to three-set capacity for enumeration [14,15] and the mean size of circles in working memory [16,17].

## 3. Experiment 2

Experiment 2 further explored the temporal decay of ensemble representation in working memory by varying the interval between the memory display and the probe display. Previous studies have shown that visual information persists in sensory memory, starting at stimulus offset and lasting for 150–300 ms [34,35]. Thus, to rule out the effect of sensory memory, the retention interval in Experiment 2 was set to 500, 1000, 2000, or 4000 ms.

### 3.1. Method

#### 3.1.1. Participants

A new group of 24 participants (15 females) took part in this experiment. Two participants were removed from the analysis because their memory accuracy or response time was beyond 2.5 SD from group means. All participants (14 females, age range of 17 to 25 years) had normal or corrected-to-normal vision, signed informed consent, and received course credit or monetary compensation.

#### 3.1.2. Procedures and Design

All the details of this experiment remained identical to Experiment 1, except for two key differences. First, the number of the set in the memory display was fixed at two, a value within the working memory capacity for ensemble representations as measured in Experiment 1. Second, the retention interval was set to 500, 1000, 2000, or 4000 ms. In the experiment, we manipulated the interval between the offset of the mask and the onset of the probe display to control the retention interval.

A one-factor (retention interval: 500, 1000, 2000, and 4000 ms) within-subjects design was adopted. The retention interval conditions were blocked, and the order of blocks was counterbalanced across participants. Using a block design brings the inherent risk of potential adaptive strategies. We compared performance differences between the first half and the second half of the block for each retention interval condition and found no significant differences related to the retention interval effect. Therefore, it could be concluded that the retention interval effect is not attributable to adaptive strategies. There were 60 trials within each condition, resulting in a total of 240 trials. The whole experiment lasted approximately 50 min.

### 3.2. Results

**Figure 3:**
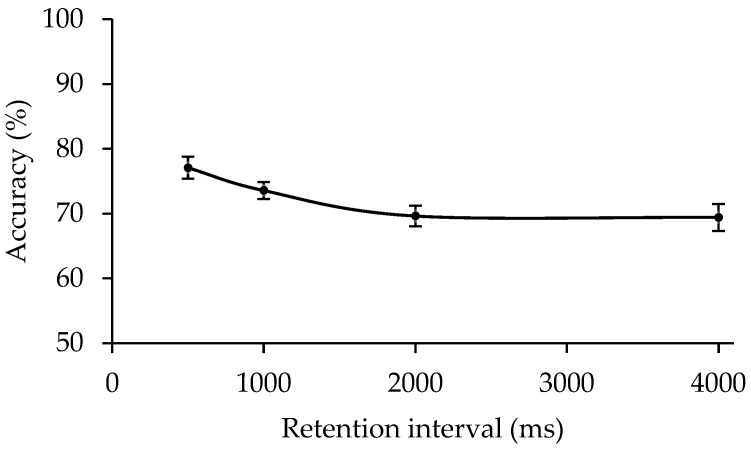
Results of Experiment 2. The accuracy is presented separately for each condition. Error bars represent SEM.

The accuracy under each condition is shown in Figure 3. A one-way repeated-measures ANOVA yielded a significant main effect of the retention interval (*F*(3, 63) = 10.502, *p* < 0.001, $\eta_{p}^{2}$ = 0.333, *BF*_10_ = 1825.596). Post hoc contrasts revealed that the accuracy was significantly higher at a retention interval of 500 ms than of 1000 ms (77% vs. 74%, *t*(21) = 2.332, *p* = 0.030, Cohen’s *d* = 0.497, *BF*_10_ = 2.029). The accuracy was also significantly higher at a retention interval of 1000 ms than of 2000 ms (74% vs. 70%, *t*(21) = 3.180, *p* = 0.005, Cohen’s *d* = 0.678, *BF*_10_ = 9.762). However, there was no significant difference in accuracy between the retention intervals of 2000 ms and 4000 ms (70% vs. 69%, *t*(21) = 0.129, *p* = 0.899, Cohen’s *d =* 0.027, *BF*_10_ = 0.225).

The decay as a function of retention interval can roughly be divided into two parts. At shorter retention intervals (up to 2000 ms), a moderate memory decline can be observed. At longer retention intervals, a level of performance is reached that is maintained without significant loss up to the longest tested interval of 4000 ms. These results indicated that ensemble representations can be stably maintained in working memory for a certain time, just like other visual materials [36,37].

## 4. Experiment 3

In Experiment 3, we manipulated the number of circles in each set to test whether the number of individuals in the set affected the memory performance of ensemble representation. Since the number of sets was not a factor discussed here, we presented participants with two sets of circles.

### 4.1. Method

#### 4.1.1. Participants

A new group of 24 participants (14 females, age range of 18 to 23 years) took part in this experiment. No participant was excluded. All participants had normal or corrected-to-normal vision, signed informed consent, and received course credit or monetary compensation.

#### 4.1.2. Procedures and Design

All the details of this experiment were identical to Experiment 1, except that the number of sets was two in all trials and the number of circles in a set was varied in different conditions. The number of circles in a set was five in half of the trials and ten in the other half.

A one-factor (the number of circles in each set: 5 and 10 circles) within-subjects design was adopted. The conditions were presented in different blocks, the order of which was counterbalanced across participants. There were 60 trials within each condition, resulting in 120 trials. The entire experiment lasted approximately 20 min.

### 4.2. Results

**Figure 4:**
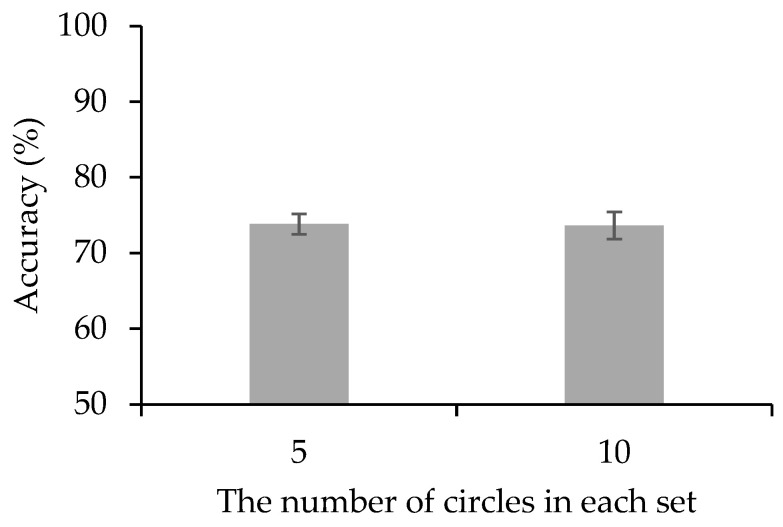
Results of Experiment 3. The accuracy for different conditions. Error bars represent SEM.

The paired *t*-test (see Figure 4) showed that there was no significant difference in accuracy between the set with five circles and the set with ten circles (74% vs. 74%, *t*(23) = 0.129, *p* = 0.898, Cohen’s *d* = 0.026, *BF*_10_ = 0.216). It provided moderate evidence for the null hypothesis, suggesting that the number of circles in the set did not affect the working memory performance of holding ensemble representation.

## 5. Discussion

The present study explored the storage mechanism of ensemble information in working memory. Experiment 1 showed that participants were able to hold mean sizes of approximately two sets in working memory. By varying the retention interval, Experiment 2 showed that there was a moderate decline in memory performance at shorter retention intervals (up to 2000 ms). However, after this initial decline, memory performance remained relatively stable without significant loss for longer retention intervals, up to 4000 ms. Experiment 3 indicated that the working memory performance of holding ensemble representation was not affected by the number of individuals within the set.

The working memory capacity in Experiment 1 was close to the value of two sets or three sets that was estimated from the previous studies [14,17,25,38]. However, the observed capacity limit was lower than the 3.5 sets when circles were spatially grouped, as reported in Im and Chong’s study [16]. This difference in capacity could be attributed to the use of different retention intervals in the two experiments. In their experiment, the retention interval was 0 s, whereas in our Experiment 1, the retention interval was 1 s. Our Experiment 2 revealed that as the retention interval increased, there was a moderate decline in participants’ memory performances, particularly during the initial 2 s of retention. This finding suggests that both retention interval and the number of sets have a combined influence on working memory performance. Future research may explore the effects and interactions of these two factors within a single experiment to gain a deeper understanding of their impact on working memory performance.

Moreover, the capacity of ensemble representations in working memory was significantly smaller than the capacity of simple objects [39]. This difference may be due to the different levels of perceptual complexity involved in processing these two types of information. Studies have shown that the estimated capacity of working memory decreased for more complex stimuli, suggesting that the complexity of the objects being remembered can play a role in determining the capacity of working memory [40,41,42]. In this study, the participants were asked to memorize the mean diameter of each set in the memory display. According to the requirement of this task, the set seemed to be reduced into a single mean. However, previous research has found that the mean representation is not a simple process of averaging individual representations into a single prototype. Instead, the results have suggested that the mean representation of a set is more complex and includes other statistical properties, such as variance, numerosity, and the shape of the underlying feature distribution [43]. This added level of complexity can make it more difficult to represent and maintain mean representations in working memory, compared to simple objects.

The current study found that the memory performance of ensembles declined during the first 2 s and remained stable in 2–4 s. This phenomenon can be explained by the rehearsal process of working memory, which refers to the active repetition or refreshment of information to maintain it in memory [44,45]. Initially, when a large amount of information is stored in working memory, the rehearsal process only partially compensates for the decay of memory traces. Therefore, memory performance declines over an increasing retention interval. However, as the decay of information stored in working memory progresses, the rehearsal process can compensate for the decay of memory traces, allowing performance to reach a stable level. This implies that memory accuracy can be maintained at a constant level regardless of the duration of the retention interval. Thus, the observed phenomenon of memory performance stopping its decay at around 2 s can be understood as the result of a delicate balance between rehearsal and decay mechanisms in working memory. Previous studies on working memory for objects have shown that memory representation remains relatively stable over several seconds. For example, Kikuchi [36] investigated the accuracy of the comparison of successively presented random dot patterns and found that the performance declined gradually with the increase in retention interval in the initial 4 s and remained relatively stable in 4–12 s. Vogel, Woodman, and Luck [39] investigated the effect of retention interval on the memory of color and found little decay of the memory representation in 900–4900 ms. Despite the different time points at which memory performance plateaus, the retention patterns of ensembles and objects are similar, suggesting that ensembles, like individual objects, can be robustly retained in working memory. Future research could explore whether the maintenance of ensemble representations in working memory is influenced by factors like distractions or cognitive load, providing a more detailed understanding of their stability.

Finally, we examined the influence of the number of within-group individuals on the memory for ensemble representations. Experiment 3 revealed that the memory performance for ensembles was not influenced by the number of individuals within the set. This result is consistent with findings from perceptual studies [3,10,27]. One possible explanation for this finding is that, after the formation of an ensemble representation, the visual system is capable of discarding individual item information [3]. The visual system extracts and maintains vital ensemble features while ignoring individual details, showcasing the brain’s exceptional efficiency in processing complex visual information. Additionally, in Experiment 3, we opted for sets containing either 5 or 10 circles. However, the distributed attention model proposed by Baek and Chong [46] supports the claim that discrimination performance increased at a decreasing rate with small set sizes and approached an asymptote for large set sizes, as predicted by the model. This finding suggests that varying set sizes may lead to distinct effects, warranting further investigation.

In summary, the current study showed that working memory could stably maintain mean sizes of approximately two sets for at least four seconds. Moreover, the number of individuals within the ensemble did not affect the working memory performance of holding ensembles. These findings contribute to our understanding of the limits and characteristics of ensemble representations in working memory.

## Data Availability

Data are available from the corresponding author upon reasonable request.
